# Report of the Poor Law Commissioners on Medical Charities in Ireland

**Published:** 1842-04

**Authors:** 


Art. I.—*Report of the Poor Law Commissioners on Medical Charities in Ireland.—Dublin,* 1841. 8vo, pp. 167.

This Report most fully illustrates the urgent necessity for an immediate reform of the medical charities of Ireland, and the scandalous abuses which at present exist in the constitution and administration of them. The enquiry extended over fifty-three unions, consisting of a population of 3,228,580, and occupying the cities and most populous towns of Ireland.

There are three kinds of institutions for affording medical relief to the sick poor; these are Dispensaries, Fever Hospitals, and Infirmaries. Dispensaries, which in 1839, amounted (in the whole of Ireland) to 615, are supported partly by subscriptions, partly by county grants and a portion of the fines imposed at petty sessions. The amount from the former source (subscriptions) was, in 1839, £34,604, for the whole island; from the latter, £34,080.

The Report proves a variety of the grossest defects and abuses connected with Dispensaries; some of which relate to the modes of support, the distribution or allocation, and the management of these charities; others, to the manner in which the medical officers are appointed and in which they perform their duties. As regards the former class of evils, it is shown that many large landed proprietors and wealthy persons subscribe nothing to the support of Dispensaries; that others, who do subscribe, make it a condition that all their tenants indiscriminately shall receive gratuitous medical relief, although many among them may be perfectly competent to pay for the same; that, in other cases, farmers, possessing 200 acres of land, and otherwise in the most easy or even affluent circumstances, obtain gratuitous medical attendance for themselves and families; and on this condition alone are their pitifully mean and meager subscriptions paid up. A twofold evil arises from the above state of things: while the rich tenantry of a landlord who subscribes, receive gratuitously that medical aid which they are well able to pay for, the poor tenantry of a landlord who contributes nothing, are often debarred from that relief to which they are entitled according to every principle of humanity as well as to the original design and the actual chartered provisions of all these charities, in which it is expressly laid down that the destitute sick shall be the only participants of their advantages. (Report, pp. 3, 4.)

Then, as regards the distribution of Dispensaries, and their proportion to the population of the respective districts in which they are placed, the greatest possible inequality prevails. While in the county of Dublin there is one dispensary to 6,286 inhabitants, in the county of Down there is only one to 23,486 persons. The disproportion seems sometimes to be still greater, for at p. 9, mention is made of "a poor tract, standing equally or more in need of relief," where "there is only one (dispensary) to a population of 33,000." It is also shown that the establishment of many dispensaries is owing to views of private interest on the part of medical officers and their friends: that these charities are often most inconveniently situated, being far removed from the more densely-peopled part of the districts, the medical wants of which they profess to supply.

As regards the modes of managing dispensaries and the way in which the medical officers, in many instances, discharge their duties, deficiencies and irregularities of the most serious sort prevail. In some cases monthly or quarterly meetings of the committees of management, or of the subscribers, are held, for the purpose of examining into the administration and expenditure; but in other cases no such meetings are ever held, and the treasurer and the medical officer (the former of whom trusts greatly or implicitly to the report of the latter) are the sole managing authority. Such a state of matters obviously paves the way to the greatest abuses and neglect.

With respect to the medical officers in many of these institutions, and the manner in which they execute their important trust, the Report furnishes a lamentable picture. In one instance a dispensary, supported by contributions, and to which the presenting authorities grant the county funds, has for its officer an individual who has no legal qualification as physician, surgeon, or apothecary (p. 3.) The following are a sample of the imperfections existing and tolerated under the present system:

"28. The medical officer does not practise midwifery, although his district is remote from the residence of any practising accoucheur. He is ignorant, whether, during 20 years, any injury has arisen from his non-acquaintance with this branch of his profession.

"29. The attending physician states, that he visits a certain number of cases in the course of the year; usually pays but one visit to each, no matter what the disease may be; even although it were fever of a bad typhoid character, he would deem a second visit rarely necessary.

"30. The medical attendant partly resides, and has a dispensary, in one county; he attends another once a week, at a distance of eight miles, in an adjoining county; his family reside in a third county, eight or nine miles further off. He is unable to say if fever has prevailed to any extent in any part of his extensive district, except in that which is near to the first-named dispensary, contiguous to which he holds a large farm.

"31. The medical officer of the institution is non-resident, and the duties, from this and other causes, are not performed satisfactorily. Certain influential persons establish a second dispensary in the same district, and appoint another medical man to attend it, who is also non-resident. Thus a district which stands in need of, and could well support one resident medical practitioner, is left without any, and subject to the inconveniences arising from this defective arrangement.

"32. The subscribers are the personal friends of the medical officer, who, although he keeps a registry of cases, yet gives the treasurer a return of the number stated by him to have been relieved; which return is laid before the presenting sessions and grand jury, and leads to the supposition that the relief afforded far exceeds that which is really given." (Report, pp. 8, 9.)

In various parts of the Report it is shown that the stocks of medicines at not a few of the dispensaries and hospitals examined by the commissioners, were of the most wretched quality and preparation, although, no doubt, this circumstance was, in some instances, to be attributed to the inadequate remuneration allowed to the medical officer. And here we may notice that, while in some cases the salaries of the medical officers appear sufficient for the amount of duty performed by them, in others the sums allowed these gentlemen are utterly inadequate as fair compensation for their toil.

*Fever Hospitals and Infirmaries.* Many of the objections just now stated to the existing constitution and arrangements of the dispensaries of Ireland, apply equally to the Fever Hospitals and Infirmaries : such as the unfeeling neglect of many large landed proprietors to subscribe to them; the inconveniences arising from these charities not being eligibly allocated; their inadequacy, in respect of number and extent of accommodation, to the wants of the sick poor; the occasional non-residence of the medical officer, as his being at an unsuitable distance ; the careless and inefficient manner in which the duties devolving on the medical officers are occasionally discharged. On all these the Report furnishes full and, as it appears to us, very correct and impartial information ; and we recommend any of our readers particularly interested in the enquiry to the document itself.

The Report advocates the new arrangement and more ample distribution of the charities of Ireland, not only as a measure of obvious humanity, but as a powerful means of checking pauperism; since, by the fact of heads of families, &c. being disabled by disease, protracted much beyond the time it would last under efficient medical treatment, all dependent on these heads of families are reduced to indigence; and thus, in addition to the evil now stated, large masses of the Irish community are predisposed to pestilential disease.

The remedial measures (p. 24) suggested by the commissioners are confined to dispensaries and fever hospitals, it being already provided by act of parliament, that infirmaries " may be supported (p. 18) by county and parliamentary funds, altogether irrespective of any subscriptions or donations." It is proposed that dispensaries and fever hospitals be provided for out of the poor rates, in order, at once, to ensure their sufficient maintenance, and equalize the burden of supporting them by throwing it on all members of the community : that each dispensary and fever hospital district be accurately defined; that the direction and management be of a popular character, in order to give the public an interest in the institutions. The heads of a proposed bill for carrying out these and other important objects connected with the due maintenance and administration of the charities of Ireland, are given at p. 33, and seem to include all that is necessary for attaining in the amplest manner that contemplated end.

We have only to add, that the conviction appears to be very general in Ireland, that the only efficient mode of securing due medical relief to the sick poor of the country, is by connecting the fever hospitals and dispensaries with the poor rates.

A variety of statistical tables and reports fill up the volume.

